# A New Wearable System for Home Sleep Apnea Testing, Screening, and Classification

**DOI:** 10.3390/s20247014

**Published:** 2020-12-08

**Authors:** Alessandro Manoni, Federico Loreti, Valeria Radicioni, Daniela Pellegrino, Luigi Della Torre, Alessandro Gumiero, Damian Halicki, Paolo Palange, Fernanda Irrera

**Affiliations:** 1 Department of Information Engineering, Electronics and Telecommunications, Sapienza University of Rome, 00184 Rome, Italy; federico.loreti@ayes.it (F.L.); fernanda.irrera@uniroma1.it (F.I.); 2 STMicroelectronics, Agrate Brianza, 20864 MB, Italy; valeria.radicioni@st.com (V.R.); luigi.dellatorre@st.com (L.D.T.); alessandro.gumiero@st.com (A.G.); damian.halicki@st.com (D.H.); 3 Department of Public Health and Infectious Diseases, Sapienza University of Rome, 00185 Rome, Italy; daniela.pellegrino@uniroma1.it (D.P.); paolo.palange@uniroma1.it (P.P.)

**Keywords:** sleep apneas, wearable devices, domestic screening, photoplethysmography, apnea type, accelerometer, signal fusion

## Abstract

We propose an unobtrusive, wearable, and wireless system for the pre-screening and follow-up in the domestic environment of specific sleep-related breathing disorders. This group of diseases manifests with episodes of apnea and hypopnea of central or obstructive origin, and it can be disabling, with several drawbacks that interfere in the daily patient life. The gold standard for their diagnosis and grading is polysomnography, which is a time-consuming, scarcely available test with many wired electrodes disseminated on the body, requiring hospitalization and long waiting times. It is limited by the night-by-night variability of sleep disorders, while inevitably causing sleep alteration and fragmentation itself. For these reasons, only a small percentage of patients achieve a definitive diagnosis and are followed-up. Our device integrates photoplethysmography, an accelerometer, a microcontroller, and a bluetooth transmission unit. It acquires data during the whole night and transmits to a PC for off-line processing. It is positioned on the nasal septum and detects apnea episodes using the modulation of the photoplethysmography signal during the breath. In those time intervals where the photoplethysmography is detecting an apnea, the accelerometer discriminates obstructive from central type thanks to its excellent sensitivity to thoraco-abdominal movements. Tests were performed on a hospitalized patient wearing our integrated system and the type III home sleep apnea testing recommended by The American Academy of Sleep Medicine. Results are encouraging: sensitivity and precision around 90% were achieved in detecting more than 500 apnea episodes. Least thoraco-abdominal movements and body position were successfully classified in lying down control subjects, paving the way toward apnea type classification.

## 1. Introduction

In the last decades, the development of microelectronics was powered by Moore’s law, with the focus on integrated circuit densification and aggressive bit integration. However, as of today, the interest is parallelly growing toward the “more than Moore” paradigm, which is based on the integration of Complementary Metal Oxide Semiconductor (CMOS) technology, radiofrequency (RF) analog and power supply circuits, and a variety of miniaturized sensors in the same chip. In the specific area of medicine, this trend gave birth to the “Health 4.0” revolution, which is characterized by the adoption of electronics components (preferentially wearable and/or implantable) and Information Communication Technology (ICT) to assist medical diagnoses directly from the domestic environment, further promoting the strategy of telemedicine [1,2,3,4,5,6]. The research in this field is skyrocketing thanks to the enormous benefits that the synergy between ICT and medicine can provide, together with the removal of distances and barriers between patient and doctors and with the adoption of remote data analysis. These benefits span from reduced time spent in hospital, with consequent reduction of costs and optimized personnel employment, to long monitoring times in free-living conditions, thus improving the patient life quality [7]. There are already many examples of this revolutionary synergy on the market, such as shoes with footbeds for gait analysis [8], fall assessment patches [9], body area networks for postural sway evaluation [10], as well as active devices as the diabetes customizable pump, approved by The Food and Drug Administration (FDA) in 2019 [11,12,13]. Wearable electrocardiogram (ECG) and blood pressure monitors are integrated in commercial products such as smart health watches, and they are used for self-monitoring and self-empowerment, which are other key points of Health 4.0 [14,15]. All these devices allow for a more precise diagnosis and customization of the therapy. For all these reasons, telemedicine is considered one of the key components for improving the health and wellness of the population in the next few years [7].

In this work, we focused on a group of pathologies named sleep-related breathing disorders (SRBD), which are characterized by recurrent episodes of pause (apneas) or reduction (hypopneas) of breathing during sleep that last at least 10 s. In the first case, we have an absence or a 90% reduction of the airflow; in the second case, the airflow is reduced by 30% and is associated with a 3% oxygen desaturation in blood or an arousal (alternatively, a 30% reduction with 4% desaturation) [16]. There are five types of sleep-related breathing disorders, according to the third edition of International Classification of Sleep Disorders: the most common is obstructive sleep apnea (OSA), which consists in a physical obstruction of the upper airways; the second type, central sleep apnea (CSA) syndromes, are due to a transient loss of neural output to the respiratory muscles; sleep-related hypoventilation disorders; sleep-related hypoxemia disorder; isolated symptoms; and normal variants [17]. Apneas could be of obstructive or central origin. A third type of apnea, called mixed, is a combination of the first two: the apnea starts as central and then becomes obstructive. OSA affects 1% to 6% of adults and 2% of children. The most common predisposing factor is obesity [18]. Other risk factors are features that can narrow airways and decrease muscle tone, such as anatomical abnormalities (micrognathia, hypertrophy of uvula and soft palate, macroglossia) or functional factors (drugs and alcohol) [19]. A particular type of sleep apnea, called positional OSA, manifests only in a specific sleeping position, usually the supine one. CSA affects less than 1% of subjects [20] and is characterized, in contrast to OSA, by apneas and hypopneas without thoracic and abdominal effort. The most relevant risk factors for central sleep breathing disorders are cardiovascular disease (CVD) and the chronic use of opiods. People who suffer from SRBD, especially OSA, complain of restless sleep, excessive daytime sleepiness, cognitive deficit, and daytime sleep attacks. This may bring to a major risk of driving or work-related accidents. Moreover, OSA is associated with CVD: if not properly treated, people suffering from OSA are more exposed to systemic arterial hypertension, myocardial ischaemia, and cerebrovascular diseases. Even sudden cardiac death could occur in the case of a severe pathology [18,21]. OSA and CSA are two distinct pathologies characterized by different therapeuthic strategies, so classifying the type of apnea is fundamental for diseases management. The most used intervention for OSA is the continuous positive airway pressure (CPAP), which opens the collapsed airways and guarantees their patency. Surgery can be a solution in case of anatomical anomalies [19,22]. For people affected by CSA, therapeuthic options are nocturnal supplemental oxygen, adaptive servo-ventilation, bilevel positive airway pressure (BPAP), and acetazolamide. The diagnosis of SRBD is still a challenge [23]. Up to date, the gold standard to diagnose SRBD is the attended polysomnography (PSG), which consists in recording signals during the night in a sleep laboratory to recognize sleep stages, monitor cardiac activity, and respiratory function [24]. It determines the severity of the disease and evaluates all the sleep disorders [16]. PSG suffers from some limitations that restrain its use. It is a cumbersome, obtrusive, labor-intensive, and expensive examination that has limitations such as night-by-night variability [25]. Consequently, there is often a tendency to use simpler tests such as the type III home sleep apnea testing (HSAT), which measures limited cardiopulmonary parameters (airflow, thoracoabdominal bands, and oximetry) and does not include sleep staging. Currently, the American Academy of Sleep Medicine (AASM) recommends home diagnosis with portable monitoring devices in patients with a high pre-test probability of moderate-to-severe OSA but no significant comorbidities [26]. Although HSAT can be performed at the patient’s home, it requires high patient collaboration due to composite equipment and could show suboptimal accuracy compared to in-laboratory measurements [27,28]. The huge demand for PSG supports the use of alternative approaches, possibly providing unobtrusive and long-term evaluations [29,30]. On the basis of these considerations, it is evident that the possibility of using wearable devices for the domestic screening and subsequent follow-up of SRBD would be a breakthrough.

In this paper, we proposed an unobstrusive wearable wireless device, called MORFEA, for the domestic pre-screening of SRBD. MORFEA seems potentially able to detect apnea episodes, detect thoraco-abdominal movements associated to OSA, grade the severity of SRBD, identify the patient and head position during sleep. The device is comfortably positioned on the nasal septum. MORFEA uses integrated photoplethysmography (PPG), an optical technique with red and infrared light sources, to analyze variation in arterial blood volume [31], and a tri-axis accelerometer, based on MicroElectroMechanical (MEMS) Technology integrated on silicon, to analyze movements. MORFEA records and transmits data in streaming mode via Bluetooth Low Energy to an external elaborator. Data can be observed in real time while acquiring. Positioning MORFEA on the nasal septum is a strength, since it allows for maximum PPG sensitivity to airflow modulation and for an excellent sensitivity of the accelerometer to thoraco-abdominal movements at the same time. Algorithms were developed for apnea detection (using PPG signal) and classification (using PPG and inertial signal fusion) and body position identification (using inertial signal). The algorithm performances in apnea detection have been tested on a hospitalized patient. The patient was wearing our integrated system on the nose and was contemporarily subjected to the AASM recommended type III HSAT. Results are extremely encouraging: with a sample of more than 500 apnea episodes, MORFEA exhibited sensitivity and precision in apnea detection around 90%. The method for apnea-type classification, based on the concomitant detection of airflow reduction (with PPG) and repeated thoraco-abdominal efforts (through the accelerometer), in those intervals where the PPG is detecting an apnea was validated on control subjects, due to unavailability of the accelerometer at the time of the patient test.

## 2. State of the Art of Wearable Wireless Devices for SRBD

Research on the use of wearable devices in sleep apneas has been growing rapidly in the last years. The monitored parameters and the strategies employed are many and various.

Actually, a first classification of those devices proposed in the literature should be made between the wire-free and the wired ones. Of course, all the devices including ECGs belong to the wired category, and for this reason, in our opinion, they are only partially unobtrusive (even holters, which are portable, have cables for the ECG electrodes). On the other hand, to the best of our knowledge, only devices using ECG or abdominal/thoracic trackers were demonstrated to be able to classify apnea episodes. For example, Surrel et al. developed a wearable system that is able to monitor obstructive sleep apneas by the means of a single ECG channel, reporting an accuracy of classification of 88.2% [32,33]. Ben Azouz et al. tested The Equivital LifeMonitor, a commercially available device, against a standard polysomnography. The LifeMonitor is a garment, worn on the chest, equipped with an ECG, accelerometer (for the body position), and a piezoresistive respiration sensor. The study shows that even if more signal assessment and processing are needed, it is possible to use the wearable sensor garment as an in-home screening system to observe and monitor sleep apnea episodes [34].

In addition to those using ECG, there are many other wearable devices monitoring movements or other physical and physiological parameters. Lin et al. realized a piezoresistive flexible band monitoring the abdominal and thoracic movements and developed a non-harmonic model able to obtain a sensitivity of about 82% [35]. Hafezi et al. used an accelerometer to record tracheal movements of patients and then implemented a deep learning algorithm to estimate sleep apnea severity [36]. Milici et al. developed a wearable system consisting of two sensors: a thermistor placed close to the nose, which detects the changing in airflow during breathing, and a galvanic skin response sensor, which measures the conductivity of the skin. The information is extracted by a low power microcontroller, which calculates the respiration rate, time of apnea, and the activation of the autonomic nervous system. The study proved a strong correlation between apneas and the autonomic nervous system activation, as well as the usefulness of galvanic skin response sensors in apnea detection [37]. Puri et al. developed a mask called ARAM that is able to monitor sleep patterns by the means of a pressure sensor, a microphone, an accelerometer, and a temperature sensor [38]. VOCNEA detects apnea and hypopnea by using gas sensors [39].

Another signal widely used to detect apneas with wearables is PPG [40]. The large diffusion and great development of this technique are due to its low invasiveness, good performance, and high flexibility. Jin Choi et al. developed a platform for obstructive sleep apnea detection by the means of a wearable “bracelet” with ECG and an integrated PPG sensor from which $SpO_{2}$ is extracted [41]. Gutta et al. proposed a more statistical approach to the detection, combining $SpO_{2}$ and heart rate data acquired from databases with mathematical models of the cardiorespiratory system and then performing the detection with a likelihood ratio test [42]. Jeon et al. combined a bio-cradle equipped with a nasal tube (respiration sensor), an accelerometer, and a finger PPG sensor with a band worn on the wrist, that was able to provide scheduling and time synchronization to the mentioned sensors. By the means of $SpO_{2}$, calculated from the finger-acquired PPG, the system detects sleep apneas and determines their severity grade. If this grade raises beyond a certain threshold, the device communicates with an external bluetooth speaker that alerts and wakes up the patient. The device works in real-time mode [43]. Arulvallal et al. developed an apnea-monitoring wearable device able to acquire heart rate, $SpO_{2}$, and blood pressure with a good correlation with the same parameters from polysomnography, especially for moderate and severe OSA cases [44].

Glos et al. compared the tracheal sound analysis for apnea severity estimation with the oronasal thermal airflow sensor, which is the first recommendation of the American Academy of Sleep Medicine (AASM), obtaining a sensitivity of 93% in apnea detection if combined with a nasal pressure sensor [45]. This is pointed out also by Penzel et al., who confirm that tracheal sounds can play a significant role in detecting sleep related disturbs [46]. Nam et al. analyzed sleep quality by the use of a pressure sensor (for heart rate variability) and a tri-axis accelerometer, including the detection of sleep apneas [47]. Another device worth mentioning is the WatchPat, which is a watch device validated for OSA detection that makes use of a traditional pulse-oximeter to measure peripheral arterial tone measurement [48].

The system proposed here detects the number and timing of the apnea episodes during nocturnal sleep, classifies the apnea type, and contemporarily records the patient body position. It is comfortably positioned on the nasal sept and is wire-free, since it uses PPG and an accelerometer integrated on silicon together with a Bluetooth Low Energy and a battery. It operates transmitting data to an external elaborator in streaming, so that the data processing is performed off-line.

## 3. MORFEA Hardware Description

MORFEA is the name of the hard device proposed here for studying the apneas. Being a wearable device, one of the main targets of MORFEA is being comfortable and unobstrusive. Furthermore, MORFEA transmits collected data in streaming mode for the whole operating time, while showing on screen the acquired signals in real time. Another requirement is the battery lifetime, since MORFEA must be active for the entire nocturnal sleep period. Given these constraints, the design of the hardware led to a restricted choice of the integrated components.

The device includes a PPG sensor with two light-emitting diodes (LEDs), a tri-axis accelerometer, a Bluetooth Low Energy system on chip, a voltage regulator, and a battery (plus other unused sensors). MORFEA transmits data via Bluetooth to a PC supporting Bluetooth 4.0. Otherwise, a Bluetooth 4.0 dongle, connected to the PC via USB, can be used. A dedicated app on the PC allows to start, pause, resume, or stop the acquisition and to set the sampling frequency individually for every sensor on the device. In our case, only the red and infrared PPG, along with all the three accelerometer channels, were enabled. Other sensors are embedded but not used in this work. The acquired data do not undergo any processing before being transmitted to the PC, where a dedicated script reorders the samples according to their timestamps and plots them on the screen in their raw state. After this step, data can be processed by the detection algorithm. This counts for each signal, both the PPGs and the three accelerations. Therefore, all the analysis is performed off-line.

BlueNRG-1. BlueNRG-1 is a very low power Bluetooth Low Energy system on chip produced by STMicroelectronics. It embeds a Cortex M0 to run the user application code and includes 160 kB of programming Flash memory, 24 kB of static RAM memory with retention (divided in two banks of 12 kB) and a Serial Peripheral Interface (SPI), a Universal Asynchronous Receiver-Transmitter (UART), and Inter-Integrated Circuit (I²C) standard communication interface peripherals. An analog–digital converter enables communications with analog sensors and battery level reading, while a digital filter allows processing a pulse density modulation stream.

**Figure 1:**
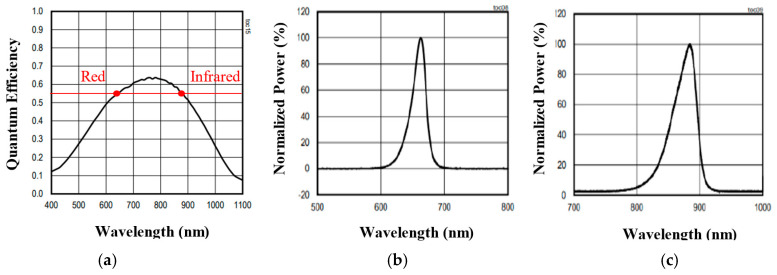
Photodiode Quantum Efficiency (**a**), Red (**b**) and Infra-red (**c**) light-emitting diode (LED).

MAX-30102. MAX-30102 is the PPG sensor embedded in MORFEA. It is produced by Maxim Integrated. It is an integrated pulse-oximetry and heart rate monitor module. PPG uses basic optoelectronics devices. It needs a light source to enlight the skin tissue and a photoreceiver to monitor transmitted or reflected light in order to visualize the blood variations. MAX-30102 includes red and infrared LEDs, optical elements, and low noise electronics that include an ambient light rejection. Red and infrared spectra of the LEDs, along with the photodiode quantum efficiency, are shown in Figure 1. The power supply is divided into two parts; MAX30102 operates with 1.8V, and LEDs have a separate power supply of 3.3 V. Communication with the Bluetooth system is through a standard that is I²C compatible. This device is specifically designed for applications where the compact size and the low-power consumption are strict requirements [49]. On July 2020, MAX-30102 was approved by the FDA for sleep apnea validation in home-based tests [50].

**Figure 2:**
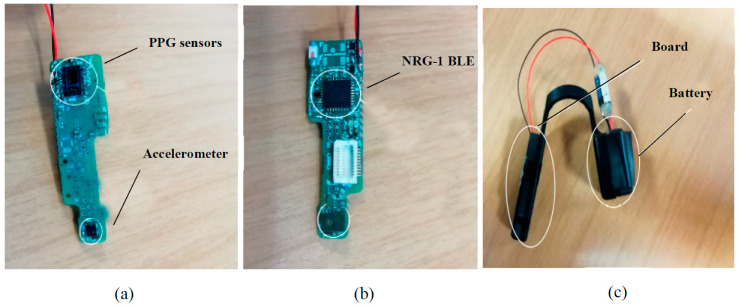
MORFEA, view of both sides with sensors (**a**), Bluetooth Low Energy (BLE) module (**b**) and the current case with the battery (**c**).

LSM6DSM. LSM6DSM is the accelerometer mounted on MORFEA and used in the apnea type classification and body position detection. It is a system-in-package that works as a 3D accelerometer and 3D gyroscope; the sensor drains 0.65mA in high performance mode. LSM6DSM has a full-scale acceleration range of ±2\ ±4\ ±8\ ±16 g and an angular rate range of ±125\ ±250\ ±500\ ±1000\ ±2000 dps. It has a communication interface that is I²C and SPI compatible and regarding power supply, it uses 1.71−3.6V [51]. A compact and light case has been designed to be positioned on the nasal septum, without altering or disturbing the patient sleep and breathing. It is shown in Figure 2. As one can see, one of the two dimensions is much longer than the other, so that the device adheres to the nasal septum length. No electrodes or cables are used.

## 4. Methods

MORFEA has been tested on one hospitalized patient who signed the informed consent and was contemporarily subjected to the conventional type III HSAT (thoraco-abdominal bands) that enables measuring two respiratory variables (effort to breathe and airflow), oxygen saturation, and a cardiac variable (heart rate). MORFEA, as well as the type III HSAT, is not able to perform sleep staging and to detect REM phase. Since the device is intended for screening, this is a minor limitation. For the apnea detection, we used only the PPG signal in reflection mode. The sampling frequency was 50 Hz. PPG has both DC and AC components. The DC component consists of the constant part of the optical signal. Its characteristics are determined by the body point from which the signal is acquired, the average tissue absorption, the average venous blood volume, and the non-pulsatile arterial blood. The AC component contains information about variations in blood volume [52]. It mainly depends on cardiac frequency, since volume variation happens between systolic and diastolic phases of the cardiac cycle and is related to breathing.

**Figure 3:**
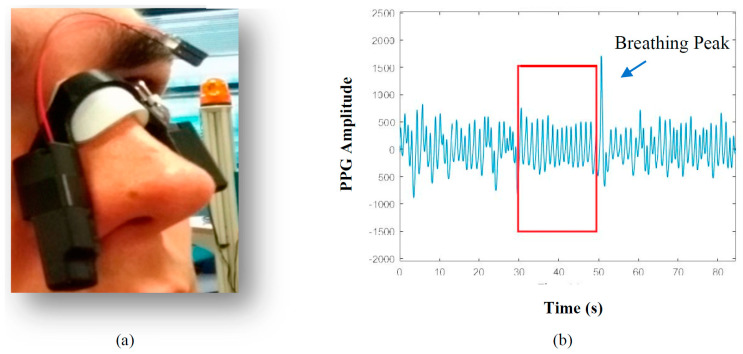
MORFEA positioned on the nose (**a**); generic filtered photoplethysmography (PPG) signal showing a central apnea (**b**).

Figure 3a is a picture of MORFEA when it is worn. During inspiration, stroke volume is reduced due to changes in intrathoracic pressure [53]. Moreover, the higher sympathetic activity that characterizes inspiration determines arterial vasoconstriction, transferring blood from arteries to big veins. During expiration, the reduction in sympathetic activity is responsible for a lower arterial muscle tone that allows blood to flow back into the arteries [54]. Positioning MORFEA on the nasal septum, we took advantage of the deepest modulation of the PPG signal during the breathing activity: indeed, during an apnea, there is a 90% to 100% reduction of airflow, so, as long as the apnea episode lasts, the PPG signal is not modulated by the breath-related movements. Actually, before choosing the nose, several other positions were tested: side neck, back neck, forehead, cheek, and wrist. As for the forehead and the wrist, the main problem was that the modulation due to breathing reduced too much with respect to the nasal septum, making the apnea detection algorithm less reliable. On the cheeks and neck, the problem was the act of swallowing, which created an artifact on the PPG signal. An example of the deep PPG modulation achieved on the nose is shown in Figure 3b, where the filtered PPG signal amplitude is drawn as a function of time during a short interval of a test. Details on signal filtering will be discussed in Section 5. An episode of (central) apnea is included in the box, before and after which the subject was normally breathing. The episode was recognized as a central apnea by HSAT. As one can see, by just filtering the signal, it appears evident that the portion in the box is much more regularly oscillating with respect to the ones outside, showing an almost flat envelope. This feature was the starting point for the development of the detection algorithm that will be discussed in Section 6. It is worth noticing that at the end of the apnea, a high breathing peak is always present, as denoted in Figure 3b. However, that peak was not considered in the detection algorithm, since its features are not always the same.

Once an apnea is detected by the PPG, there is another great advantage of positioning MORFEA on the nose. Indeed, on the nose, the integrated accelerometer can successfully detect even the least thoraco-abdominal movements, of the type manifested by patients during an obstructive apnea. This has been demonstrated by repeated tests in which control subjects performed any kind of small and wide effort implying thoraco-abdominal movements while lying down in any position. So, on the basis of these tests, we can presume that with real patients, after the apnea detection, the excellent sensitivity of the septum-placed accelerometer to thoraco-abdominal movements could be used for the type classification.

**Figure 4:**
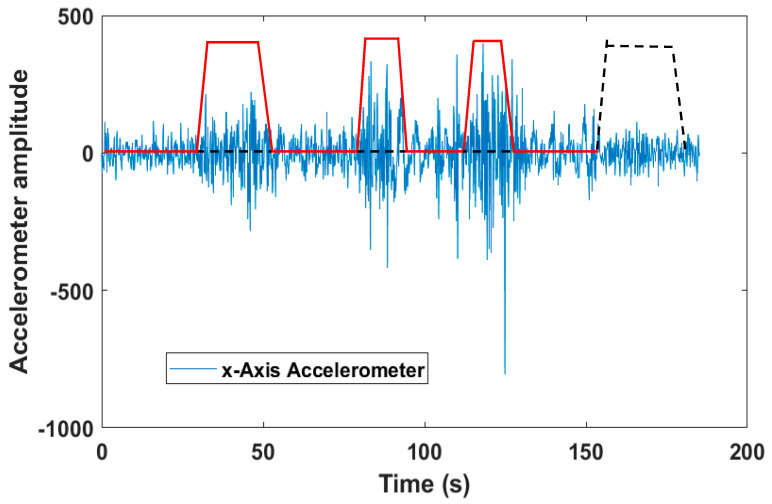
X-axis accelerometer trace: obstructive (red continuous line—flag) and central (black dashed line—flag) apneas.

We focused our investigation on one of the most important parameters related to the pathology. This the Apnea–Hypopnea Index (AHI) of a patient, which is defined as the number of events divided by the monitoring time, and it is counted in hours. It quantifies the pathology severity and is described by well-known clinic ranges [55]. In particular, AHI<5 means pathology absence, while AHI>30 implies the maximum severity. For the apnea type classification, we used a fusion of the PPG and the acceleration signals. PPG is in charge of the apnea detection, while the accelerometer is in charge of the type classification in those intervals in which the PPG is detecting an apnea. The possibility of detecting any least thoraco-abdominal movement was validated on a wide set of control subjects, because at the time of the test on the hospitalized patient, the integrated accelerometer was not available. We based this possibility on the following assumption: an episode of central apnea is not accompanied by thoraco-abuminal efforts, whereas obstructive apnea is characterized by repeated efforts and movements in the attempt to unblock the obstructed airways. Those movements propagate along the body, and the excellent sensitivity of the integrated accelerometer makes it possible to detect even the least ones. The control subjects performed a sequence of conditions of absence of airflow with and without the concomitant thoraco-abdominal movements, many times and in a different order, while lying down in different positions. The effectiveness of the inertial detection was always demonstrated. As an example, Figure 4 displays the filtered single axis accelerometer signal of a specific test, together with four flags corresponding to the PPG detection report. The accelerometer sampling frequency was 16 Hz. Details on the filtering technique will be discussed in Section 5. As one can see, the accelerometer signal under the dashed line–flag appears quite different from the signal under the continuous line–flags. So, the fusion of PPG and accelerometer signals creates a solid criterion for the apnea classification, as will be described in detail in Section 7.

Finally, regarding the detection of the body position, the raw accelerometer data were used. As already mentioned, this point can be very important if the patient suffers by positional OSA, since the obstructive respiratory events could be mitigated by an educational path aimed to taking up the correct sleep position. The algorithm is based on different thresholds assigned to the different sleep positions (supine, prone, on a side). It will be discussed in Section 8.

## 5. Signal Pre-Processing

Once transferred and stored on the PC, the signal is fed to a preliminary script that creates two vectors: one for the samples and one for their timestamps. Samples are originally acquired in binary format, so it is necessary to make them readable by conversion in the decimal format. After the conversion, samples are re-ordered by their timestamps, and the raw signal gets plotted on the screen. The whole algorithm does not work in real time as well as the preprocessing. The signal preprocessing is divided into three steps: (a) windowing; (b) artifacts removal; and (c) filtering.

 **a**  **Signal Windowing**

**Figure 5:**
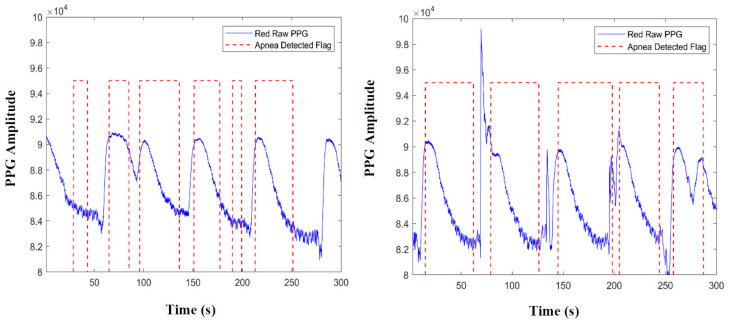
Double apnea detection prevented by the 30 s of overlap.

By design, the idea is to process one signal tract at a time: five minutes of signal are acquired, and their processing ends before the end of the acquisition of the next five. This choice follows the project constraint that limits the elaboration time, since everything is thought to be embedded on the microcontroller in the future. Another advantage that comes from windowing is adaptive amplitude thresholds, which allow setting custom thresholds depending on the morphology of the considered signal tract. The PPG signal is not constant in oscillations, so having adaptive thresholds instead of fixed ones is a real helping hand. The window length regulates the adaptive threshold optimization, since having a too short window will not allow capturing both apneas and respiration tracts. A window that is too long, on the contrary, worsens the effectiveness of an adaptive threshold and adds more computational weight. From our tests on this specific patient (with more than 500 events), five minutes is a good compromise, with the addition of a 30-s overlap between a window and the next one. This means that the last 30 s of a section coincide with the first 30 s of the next section. The overlap allows detecting apnea events that are divided between two windows, as shown in Figure 5. Only the event in the second window will be counted, preventing double detections and thus improving the AHI parameter computation.

 **b**  **Artifacts removal**

Before performing the detection itself, it is necessary to remove artifacts caused by the patient movements during the night. This code section operates on the 5-min window of the raw signal, before the filtering phase. The removal of motion artifacts is a very complex process. In the literature, we found some methods to ease this problem; one example is applying the Fourier transform on a cycle-by-cycle basis or applying the ensemble empirical mode decomposition (EEMD) method [56]. All these solutions require a lot of operations and would compromise the respiratory component of the signals. Therefore, we chose to detect and exclude the corrupted samples in order to prevent the results from being compromised. For this specific exam, motion artifacts are extremely reduced; therefore, the algorithm just identifies the corrupted parts (by searching for abrupt amplitude variations in the PPG signal) and excludes them from the analysis without any loss of information related to the apnea detection. The program does this by obtaining the average amplitude of the signal in the current window and computing the mean of the samples. Subsequently, it applies a moving window with a length of 2 s and an overlap of 1 s. The window is very short because it has to capture only the artifacts, which are usually spikes of short duration. Then, the maximum value of each 2-s length window is computed and compared with a threshold. The threshold value is obtained as:

$$T_{MC}=AVA*1.07$$

where AVA is the average amplitude of the 5-min window, MC stands for motion corruption, and 1.07 is a multiplication coefficient that has been obtained empirically, for this specific patient, after various comparisons between the motion artifacts and the normal respiration. Once these events are detected, they are saved in a vector. The apnea events detection is computed with a threshold method (described in Section 6). These thresholds are based on the 5-min long signal tract morphology. The motion-corrupted sections affect the threshold computation with higher values. For this reason, they are excluded by the thresholds computation for the apnea detection.

 **c**  **Signal Filtering**

Filtering is one of the most important phases of preprocessing. Two different filters have been designed: one for the PPG signal and the other for the accelerometer signal. In designing the filters, we aimed to maintain the cardiac (around 1 Hz) and the respiratory (around 0.4 Hz) frequencies. PPG signals acquired with MORFEA are filtered to cut off the high-frequency noise and its DC component; this is done with a bandpass filter. In order to minimize the computational weight of the algorithm, we needed a compromise between performance and filter order.

**Figure 6:**
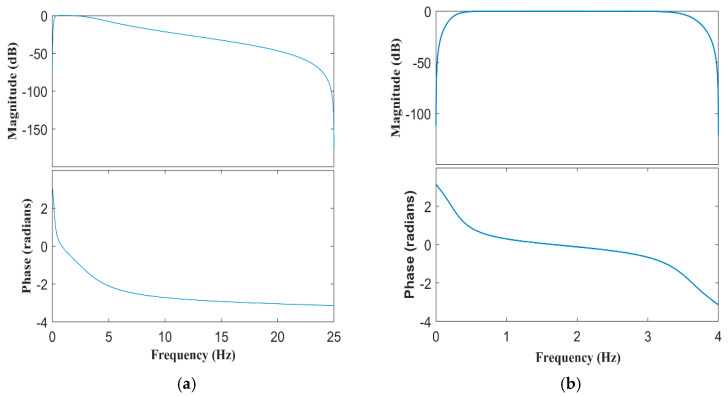
Magnitude and phase of the Butterworth filter used for PPG (**a**) and accelerometer (**b**).

The first choice concerns the filter type: since real-time filtering is not needed for this application, we chose an Infinite Impulse Response (IIR) filter. It means that the impulse response h(t) does not have a finite length over the time, in contrast with the FIR filter (Finite Impulse Response), in which time response h(t) goes to zero for t > T. The main advantage of IIR filters is the efficiency of their implementation, because they can provide a better condition of passband, stopband, ripple, and/or roll-off with a lower filter order, or rather fewer coefficients, than with FIR. A disadvantage of an IIR filter is its non-linear phase due to the constant group delay, which is a problem avoidable with FIR filters when designed with symmetric or anti-symmetric coefficients. The main problem of a non-linear phase is the variability of the time delay for the different frequencies after the filtering process. Only a few types of IIR, for example the Bessel filter, can provide a quasi-linear phase. We finaly chose an IIR filter. To include the cardiac and respiratory components and cut off high frequency noise and its DC component, a passband Butterworth filter is used. The chosen bandwidth is Δ = 0.3Hz − 3.5Hz. The coefficients of the filter are calculated in order to obtain a 4th-order filter with a passband of Δ. The filter was designed with the MatLab app “Filter Designer”, its magnitude and phase frequency responses are shown in Figure 6a. We used a technique to compensate the intrinsic non-linear phase response of an IIR filter. The signal is firstly filtered in the forward direction; then, the obtained sequence is reversed in the time domain, and it is fed back to the same filter. The obtained result provides a zero-phase filtering distortion. The transfer function is equal to the squared magnitude of the original filter transfer function and the total filter order is the double of the initial order, which means an 8th order filter. Even if the order has been doubled, this value is low if compared to the FIR filter order necessary to have the same results. For the accelerometer signal, we used a similar filter, a 4th-order Butterworth, with the same double filtering technique adopted for PPG. The only difference is the bandwidth; this time, we chose ∆ *=* 0.2*Hz* − 3.5*Hz*. The magnitude and phase frequency responses of the accelerometer filter are shown in Figure 6b.

## 6. Detection of an Apnea Episode

**Figure 7:**
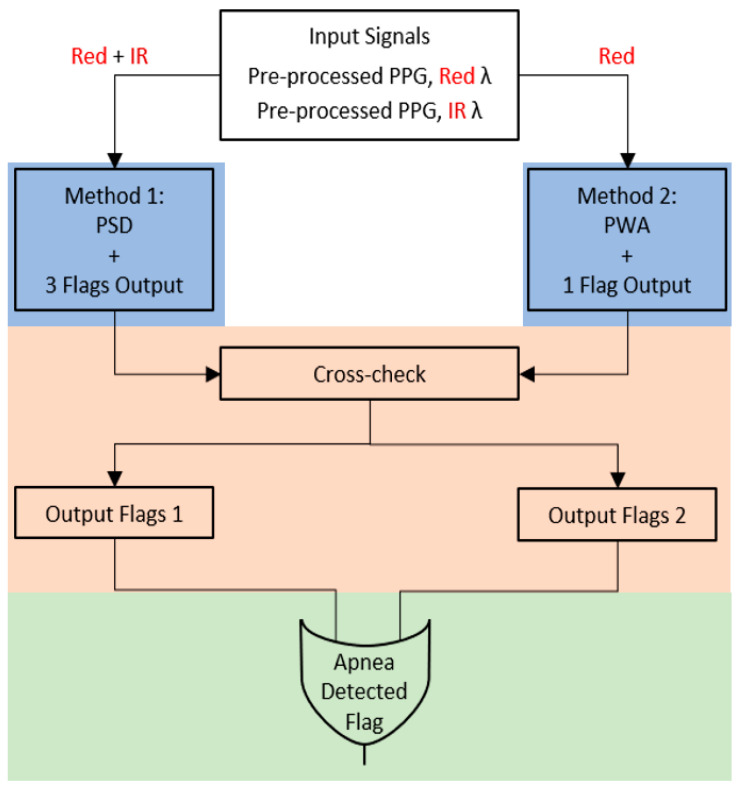
Logic scheme of the apnea detection algorithm. The “flags” are numerical feedbacks that propagate apnea detection results through the different steps of the algorith.

The main idea is to detect apnea events in different ways and with different strategies, which is followed by combining the results to obtain one final more reliable output. Since we have two PPG signals, it is possible to analyze both and check if what we saw on the first is also present in the second and vice versa. The detection algorithm is summarized in Figure 7. We implemented two strategies. The first one is based on the Power Spectral Density (PSD) of the PPG signal envelopes (left blue box in Figure 7), while the second one makes use of its amplitude, more specifically of the Pulse Wave Amplitude (PWA) signal (right blue box). Both these strategies are applied on a 5-min window at a time. The output of this step is a set of flags (flag = 1 means that an apnea event is detected, flag = 0 means normal breathing) for method 1-PSD and another flag for method 2-PWA. Then, the flags of those first sets are cross-checked to obtain just two flags, each one containing true information about detected episodes. Finally, an OR operation between the two flags gives the final overall count of apnea episodes.

 **a**  **Method1-PSD and Method2-PWA**

**Figure 8:**
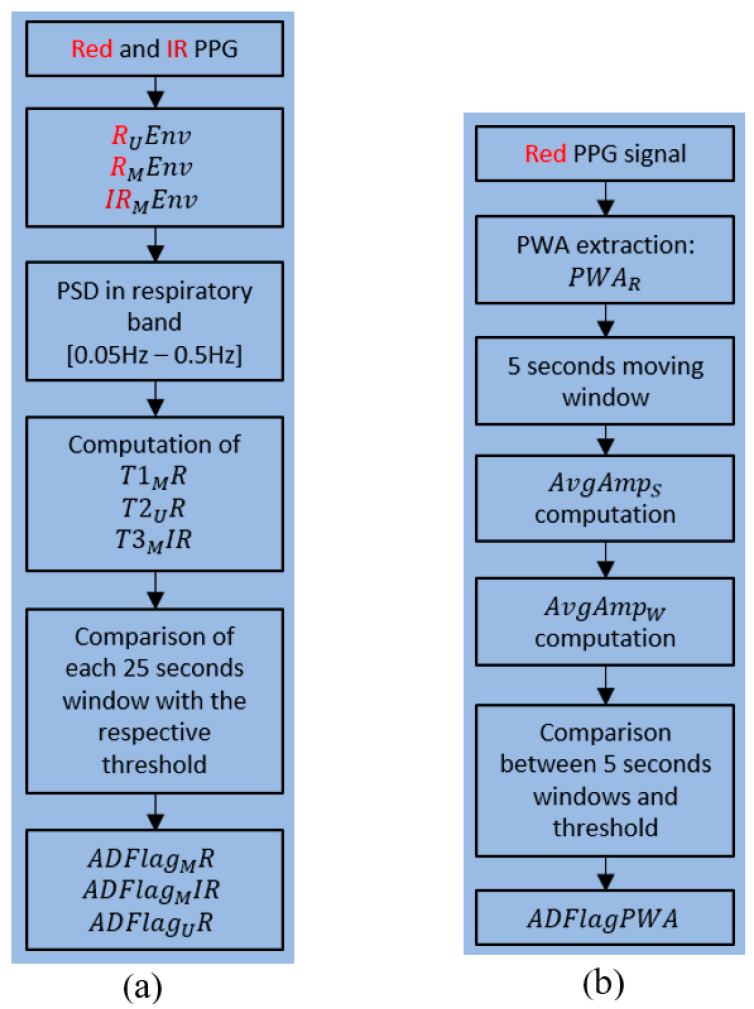
Method1-PSD (**a**) and Method2-PWA (**b**) detailed schemes. PWA: Pulse Wave Amplitude.

**Figure 9:**
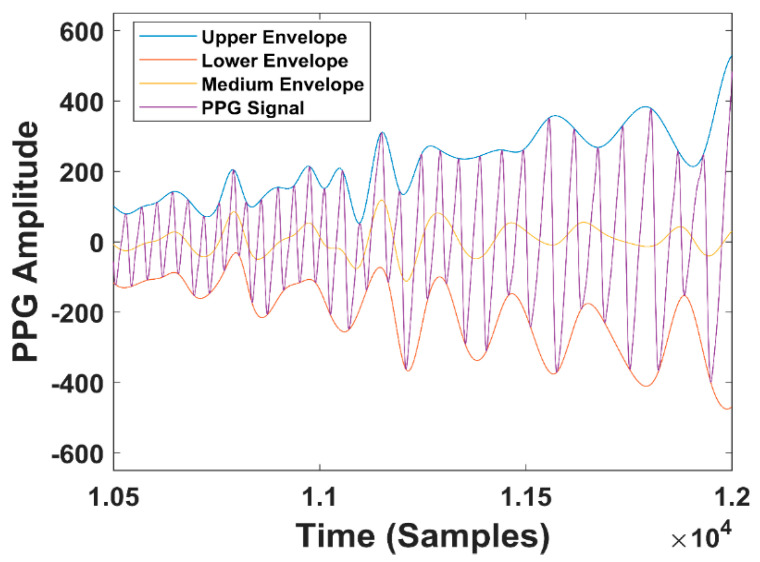
Upper, medium, and lower (not used) envelopes of the PPG signal.

Here, we describe the two strategies outlined with blue boxes in Figure 7: method1-PSD and method2-PWA. The first strategy (PSD) is described in detail in Figure 8a. First of all, we considered the Red and the IR PPG signal. The algorithm inputs (second block in Figure 8a) are the medium $\left(R_{M}Env\right)$ and upper ($R_{U}Env)$ envelopes of the Red PPG signal. The three envelopes are shown in Figure 9. We used only the upper and medium because they realize the most evident differences between breathing and apneas. We also considered the medium envelope of the IR signal ($IR_{M}Env)$. On each of the three signals (representing the breathing component), we applied a 25-s moving window with an overlap of 24 s (preprocessing in method1). This means that the moving window advances 1 s at a time, for a total of 300 windows: we computed PSD for each window in the respiratory band 0.05−0.5 Hz (third block in Figure 8a). To compute the PSD of a 25-s-long window, we used the Fast Fourier Transform (FFT) and then multiplied for the hamming window with a length equal to the length of the window. Then, the algorithm computes the power at the selected frequencies, saving the average PSD in the band for each 25-s window.

**Figure 10:**
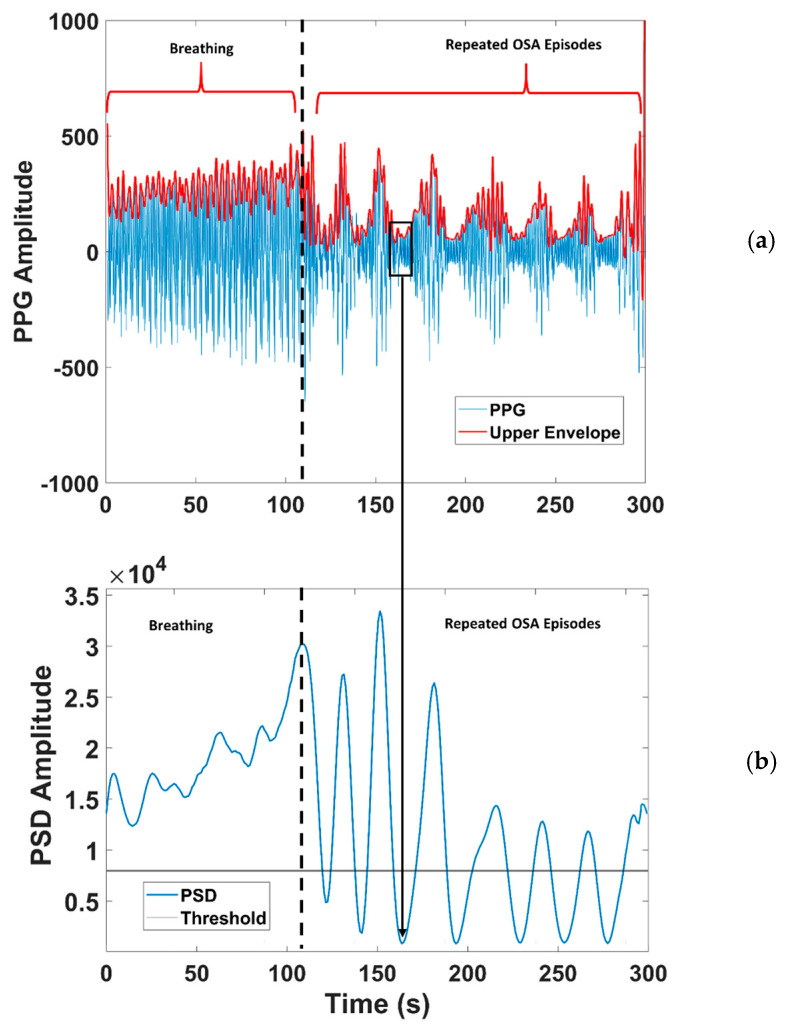
**(a**) PPG signal (blue line) and its upper envelope (red line) versus time in a 300 s window; (**b**) PSD Amplitude versus time in the same window. The black box in (**a**) highlights an OSA event approximately happening from second 160 to second 180, while the arrow shows the corresponding PSD minimum.

We obtained 300 PSD values that are averaged into a single value for each 300-s window. Given this window length, some tracts of normal respiration are guaranteed to be found (an apnea lasts 120 s as maximum). Here, we have the key working principle of the algorithm, which is made possible by the the position chosen for MORFEA. By positioning the device on the nasal septum, we take strong advantage of the movements caused by the airflow of the breathing process: the effects of these movements are significantly enhanced because the device acquires them exactly from on top of the nose. This is easily seen in the strong PPG low-frequency modulation and wide oscillations of the upper envelope that happen during breathing phases and disappear during any type of apnea. This makes sure that the PSD calculated during breathing tracts is higher than the one computed for an apnea, be it central or obstructive. This is shown in Figure 10, where we see the oscillating PPG signal and its upper envelope, along with the corresponding PSD.

**Figure 11:**
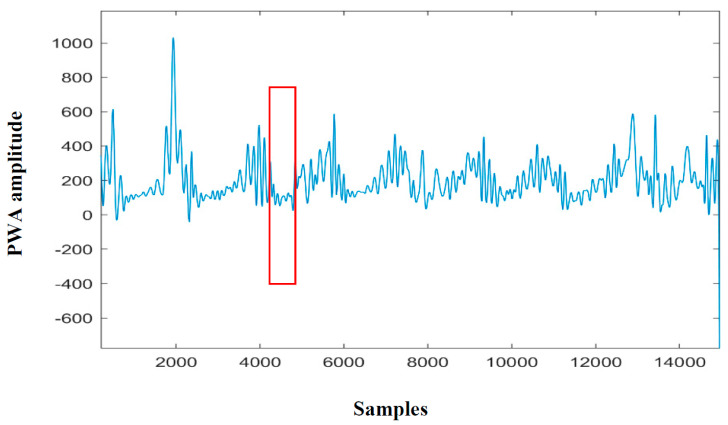
Portion of PWA signal obtained from the red PPG signal.

This means that the average PSD is always higher than the PSD of an apnea tract, so it can be used to compute a threshold for the detection. This is clearly visible in Figure 10. In the time interval 0–115 s, normal breathing is present (left of the dashed line), in the 115–300 s, seven repeated OSA episodes (minima in both plots) are present (right of the dashed line). The algorithm searches for low-power PPG sections lasting at least 10 s so that they can be considered apnea episodes. For the choice of the threshold (fourth block in Figure 8a), we saw that the raw average value cannot be directly used as the threshold because it is too high. Indeed, even some breathing tracts fell under it, possibly causing false positives. To avoid this, we scaled down the average values and defined the following thresholds, which proved to be the best choice for sensitivity:

$$T1_{M}R=0.6*APSD_{M}R$$

$$T2_{U}R=0.6*APSD_{U}R$$

$$T3_{M}IR=0.7*APSD_{M}IR$$

where *T*1*_M_R* is the threshold for the medium envelope of the Red PPG signal, *T*2*_U_R* is the threshold for the upper envelope of the Red PPG signal, and *T*3*_M_IR* is the threshold for the medium envelope of the IR PPG signal. $APSD_{M}R$ is the average PSD value of the single 300-s window obtained from the medium envelope of the Red PPG signal, $APSD_{M}IR$ is the average obtained from the upper envelope of the Red PPG signal, and $APSD_{U}R$ is the average obtained from the upper envelope of the Red PPG signal. These thresholds are not universal but rather specifically computed for every patient. This means that with more data, we will be able to adapt and customize their computation even in case of comorbities, such as Cheyne Stokes Respiration. This device calibration will be one of the subjects of the next studies. Once the thresholds have been computed, the apnea detection starts (fifth block in Figure 8a). If the algorithm detects a section of the window with the average PSD value under the threshold, and if this condition is verified for 10 s in a row (10 consecutives windows), the apnea is detected. The output of this code section (sixth block in Figure 8a) is given by three flags with a value of 0 or 1 at each second, respectively, for normal respiration or sleep apnea. The first flag is $ADFlag_{M}R$, which is obtained from the detection performed on the medium envelope of the red PPG signal, the second is $ADFlag_{M}IR$, which is from the medium envelope of the IR PPG signal, and the third is $ADFlag_{U}R$, which is from the upper envelope of the red PPG signal. The three flag signals are independent from each other (each detection is not related to the other two). The start and stop times of each apnea are also stored in this section. The detailed block scheme of Method2-PWA (right blue box in Figure 7) is shown in Figure 8b. First of all, we considered the Red PPG signal only and we used the PWA extracted from it $\left(PWA_{R}\right)$ (second block in Figure 8b). During an apnea, the respiration stops, and so do the movements of the nostrils. The power of the signal breathing component goes to zero, and since MORFEA is placed on the nose, this also reflects on the amplitude of the PPG signal. The PWA is calculated using the local maxima and minima and is composed by one sample for each maximum and minimum couple of consecutives. A portion of the PWA signal obtained from the red PPG is shown in Figure 11.

The sample amplitude is calculated as the difference between the maximum and the minimum value of the same cycle. The signal obtained as the difference between the maximum and minimum is interpolated using a cubic spline in order to obtain clean oscillations and better signal readability and refinement. It is possible to observe various decreases in amplitude, such as the one highlighted with the red box, which identifies a possible apnea event.

As with the PSD strategy, a window is used also with the PWA signal (third block in Figure 8b): the length is five seconds with an overlap of four seconds. Conversely to the PSD method, in this case, the window is much shorter. This is due to the fact that in the case of an obstructive apnea, the amplitude reduction would happen just in the first seconds, since in the following, the amplitude increases again for the chest efforts. The average PWA amplitude is computed for every window $\left(AvgAmp_{W}\right)$ for a total of 300 PWA values. At this point, each one of them is compared with the average amplitude of the entire signal section of five minutes $\left(AvgAmp_{S}\right)$. If the condition

$$AvgAmp_{S}-AvgAmp_{W}>50\;\mathrm{LSB}$$

is verified for at least five seconds, a possible apnea event is reported (sixth block in Figure 8b). Here, 50 LSB is the mean value of the distribution of the amplitude differences between an apnea event and the previous respiration tract. It is patient-dependent and fixed time by time through an automatic calibration off-line. The output of this block is the flag signal ADFlagPWA (last block in Figure 8b).

 **b**  **Flag Cross-Checking and Output Flags**

**Figure 12:**
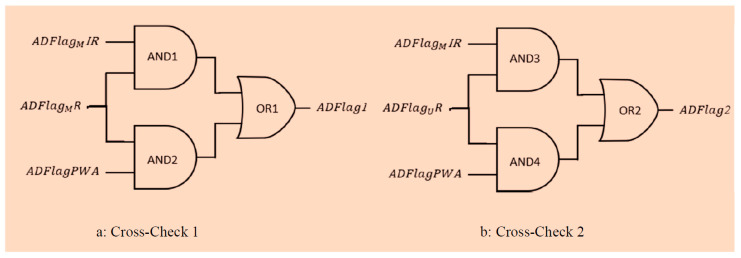
Algorithm logic gates scheme for cross-checking outputs of method1-PSD (**a**) and method2-PWA (**b**).

The four flags obtained with method1-PSD and method2-PWA are cross-checked to obtain one decisive result. For clarity, we described the corresponding pink block in the middle of Figure 7 using logic gates. The overall cross-check block is divided in two parts, as shown in Figure 12: cross-check 1 considers three flags, namely $ADFlag_{M}R$, $ADFlag_{M}IR$, and ADFlagPWA (Figure 12a), while cross-check 2 considers $ADFlag_{U}R$, $ADFlag_{M}IR$, and ADFlagPWA (Figure 12b). Both the cross-check branches trust preferencially the PSD flags comparison (AND1), in the sense that they use AND2 with the PWA flag as input only in the case that the PSD inputs do not agree (AND1 = 0). So, the first cross-check procedure described in Figure 12a verifies with AND1 for each apnea event detected in ADFlagMR if the same apnea is detected in $ADFlag_{M}IR$. If not (AND1 = 0), the algorithm compares $ADFlag_{M}R$ also with *ADFlagPWA* with AND2. ADFlag1 is the OR of the two AND operations.

**Figure 13:**
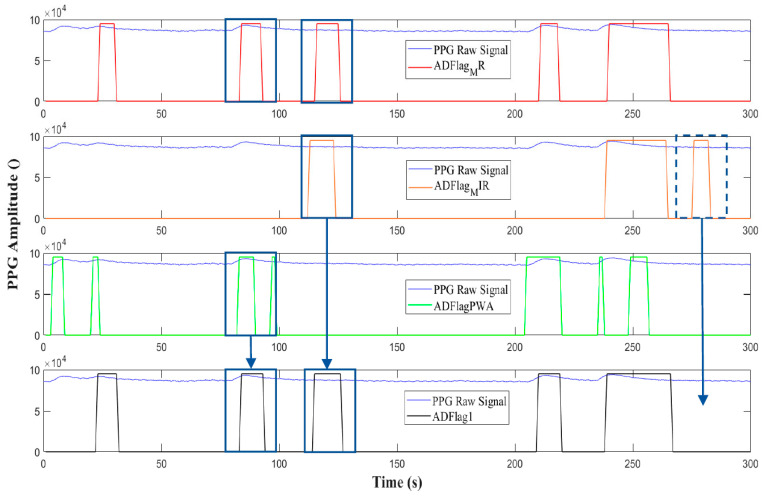
Operation of the apnea detection algorithm as an OR of two AND gates. Please note that in this case, the term “flag” represents a graphical representation of the apnea detection.

The choice of using $ADFlag_{M}R$ as the reference is due to the better sensitivity (number of events detected, which includes also false positives). This method aims to reduce the false positives, because the event has to be detected on at least two flags before being considered by the algorithm. An example of this is given in Figure 13. The event in the dashed line box is detected only in $ADFlag_{M}IR$, so the algorithm does not count it in ADFlag1. Similarly, the events in the solid line boxes are reported in ADFlag1 because they are present in at least two signals: the first event is present in $ADFlag_{M}R$ and ADFlagPWA but absent in $ADFlag_{M}IR$, while the second event is present in $ADFlag_{M}R$ and in $ADFlag_{M}IR$ but not in ADFlagPWA. Both are counted in ADFlag1. The starting instant of the event is given by the average of the start instants of the two detection flags, while the stop instant is the one used in the PSD detection method with the red PPG signal. The same process is shown in Figure 12b, with $ADFlag_{U}R$ as a reference and with ADFlagPWA and $ADFlag_{M}IR$ as cross-check signals. Similarly, an output vector called ADFlag2 is obtained. The algorithm takes into account and corrects eventual time desynchronizations among the three flag signals. Please note that in Figure 13, we use the term flag to indicate a graphical representation of the apnea detection performed by the algorithm.

 **c**  **Final Apnea Detection Flag**

**Figure 14:**
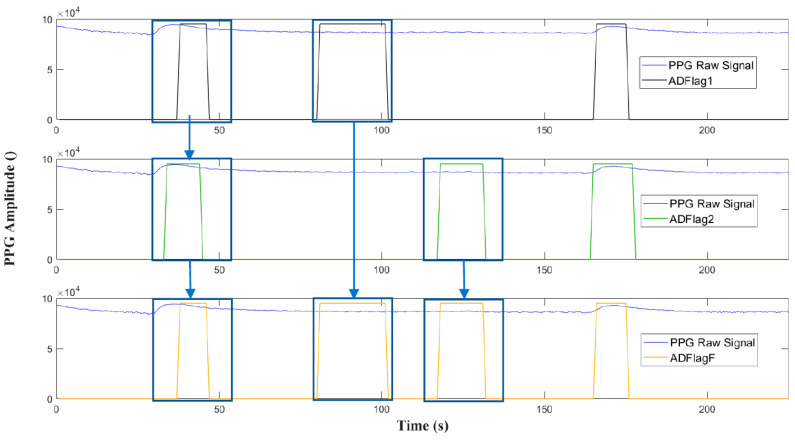
OR operation between ADFlag1 and ADFlag2 with ADFlagF as output.

Here, we describe the bottom box depicted in Figure 7 (the gray one). In order to obtain an even more comphrensive result, the OR operator is applied to ADFlag1 and ADFlag2, as shown in Figure 14. The three apnea events detected by just one between ADFlag2 and ADFlag1 are reported by the final flag ADFlagF. The algorithm takes into account and eliminates eventual apnea duplicates present in two consecutive windows, which is a common happening since many windows are truncated in the middle of an apnea event. This makes the final result more reliable.

 **d**  **Results**

The monitored hospitalized patient wore MORFEA on the nose and performed the type III HSAT at the same time, for the whole night, as per AASM recommendations. That patient suffered from a huge number of apnea and hypopnea episodes (545) of central, obstructive, and mixed type, so the statistics looks meaningful, although tests on more patients are needed to check the repeatability of the detection strategy.

MORFEA is able to detect both apnea and hypopnea events, but it classifies them under the common name of “apnea”, so the AHI generated by MORFEA and the respiratory polygraphy are comparable. Detection performances are reported in Table 1 in terms of sensitivity (defined as the ratio between the true positive events and the sum of the true positive plus the false negatives) and precision (defined as the ratio between the true positive events and the sum of the true positive plus the false positives), both referred to our standard, and AHI, reported for the respiratory polygraphy and MORFEA.

As one can see, both sensitivity and precision are excellent (around 90%), and the AHI measured by MORFEA is 104, while the value measured by the HSAT is 102, which is an extraordinary match. These performances indicate that MORFEA can be a useful device for screening and approximately grading SRBD.

## 7. Algorithm for the Apnea Type Classification

The central apnea lacks the respiratory effort, conversely to the obstructive one where the chest and abdomen make repeated efforts to take in air. We verified incontrovertibly that thanks to its excellent sensitivity, the accelerometer positioned on the nasal septum is able to detect clearly those movements propagating along the body segments, although an attenuation of the signal amplitude is obviously present. We used this criterion for the distinction between the two apnea types. The fusion of PPG and accelerometer signals is performed, since the accelerometer works only in those time intervals where the PPG is detecting an apnea. The possibility of detecting any least thoraco-abdominal movements was validated on a wide set of control subjects, because at the time of the test on the hospitalized patient, the integrated accelerometer was not available. The control subjects performed a sequence of conditions of airflow reduction or absence with and without the concomitant thoraco-abdominal movements, many times and in a different order, while lying down in different positions. The effectiveness of the inertial detection was always demonstrated. Since all the three axes of the accelerometer were deeply sensitive to these movements, we chose the one that proved to be more sensitive (x-Axis).

**Figure 15:**
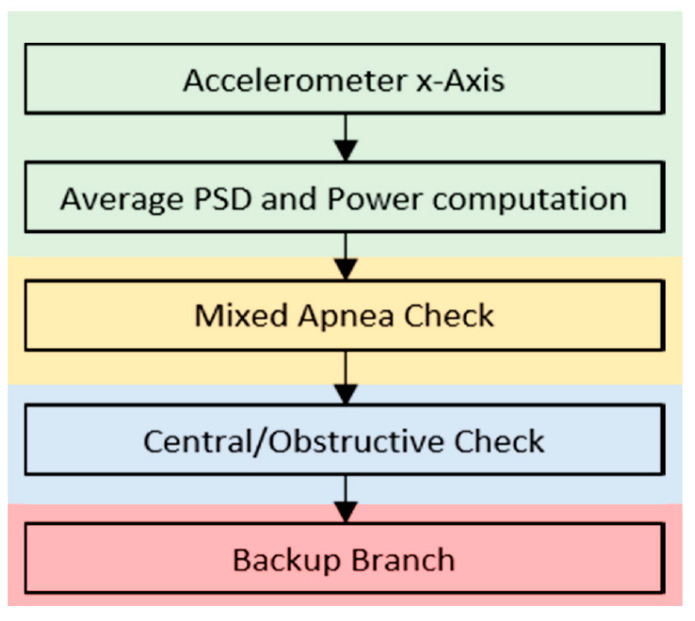
Phenotype classification block scheme.

The algorithm uses as inputs the filtered accelerometer signal and the timing of the apneas detected, so that it studies only movements during the apnea episodes. On these intervals, we applied a moving window of 25 s with an overlap of 24 s, and we computed the average PSD. This step is resumed in the first two blocks highlighted in gray in Figure 15.

**Figure 16:**
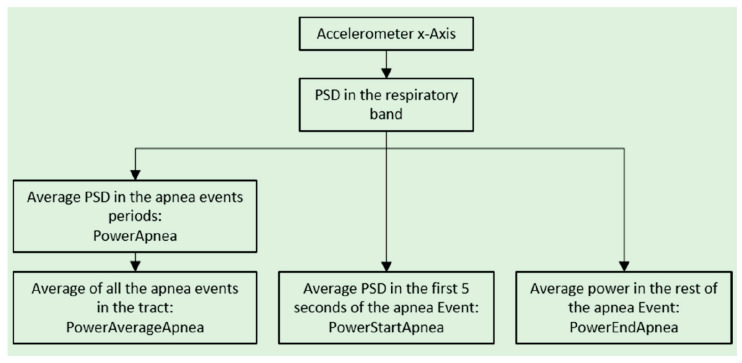
Detailed scheme of the average Power and Power Spectral Density (PSD) calculation.

**Figure 17:**
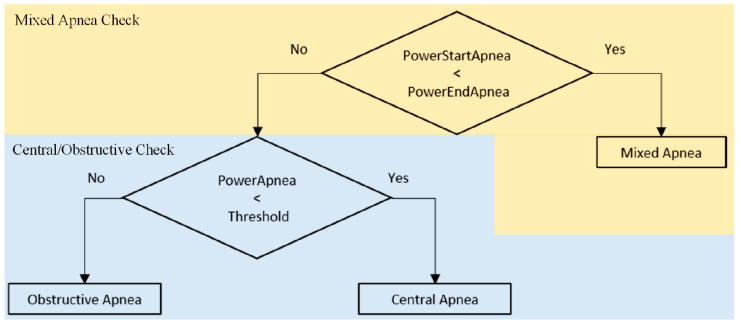
Apnea classification block schemes. Mixed (top) and Obstructive/Central (bottom) checks.

**Figure 18:**
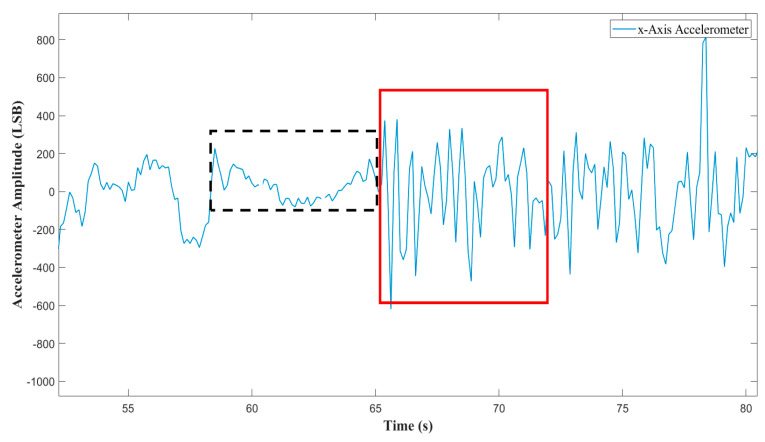
Mixed apnea morphology. Central (dashed black-line box) and obstructive (solid red) behavior.

The power computation is described below, with the aid of the scheme in Figure 16. For each apnea, we computed the PSD as the average of all the PSDs of the windows covering the event. In this way, we obtained the value called PowerApnea. Then, we averaged the PSDs from all the apnea events in the 300-s tract, obtaining the value PowerAverageApnea. For each apnea, a vector containing the PSD of the first 5 s (PowerStartApnea) and the PSD of the rest of the event (PowerEndApnea) is also generated. It is possible to create a first stage of classification using these obtained values: we can use PowerAverageApnea to compute a threshold to compare with all the apneas in the 300-s window. To classify an apnea as mixed, this strategy is not necessary, as shown in Figure 17. Applying the same criterion to mixed apneas would lead to errors, since a mixed event would be classified as a CSA. Instead, we used the values PowerStartApnea and PowerEndApnea just collected. A mixed event has the behavior of a central apnea in the first seconds and of an obstructive one in the rest, as shown in Figure 18. The amplitude of the accelerometer signal is lower in the first seconds (dashed black box) and quite higher in the remaining seconds (red solid box). The algorithm uses the ratio between the power in the first 5 s of the event (PowerStartApnea) and the power after the first 5 s (PowerEndApnea) to check if the event is mixed. The criterion used is the following: if $\frac{PowerStartApnea}{PowerEndApnea}<0.5$, the apnea is classified as mixed.

**Figure 19:**
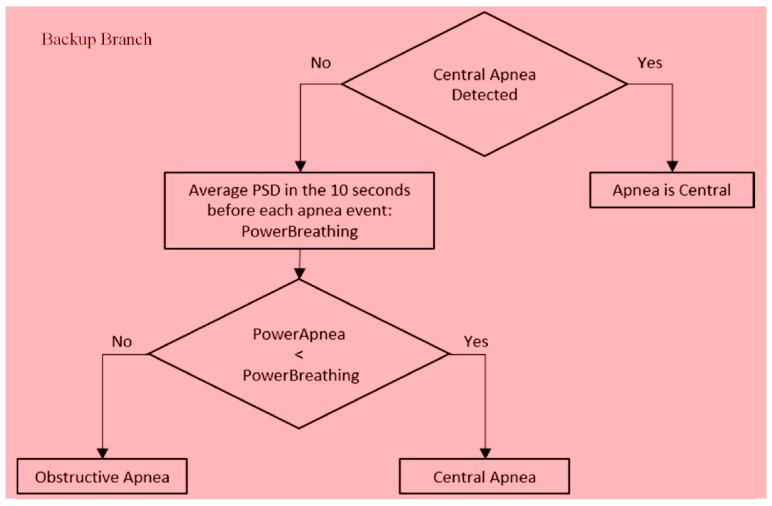
Backup branch in case of no obstructive apneas detected.

We ended up seeing that an amplitude ratio of a half is a quite reasonable value to identify the difference between the start and the end of the apnea. If the event is not classified as mixed, the algorithm decides whether it is central or obstructive, as shown by the blue block scheme in Figure 17. This happens by comparing the PSD of each apnea (PowerApnea) to the threshold and verifying the following condition: $\frac{PowerApnea}{PowerAverageApnea}<0.5$. If this is verified, the apnea is central; otherwise, it is obstructive. Sleep movements may occur at any stage. If a few events were not detected due to the overlapping of movements, there would be a slight understatement of the severity of the disease (AHI). Since Morfea will be used for screening, this is a minor limitation. Anyway, even in case of movements at the end of a CSA (arousals), the algorithm would still be able to classify it as CSA and not as an OSA, since we used mean values for the thresholds. If the patient stays still for most time of the CSA and only moves for a short time at the end, the mean PSD value would still be less with respect to an OSA with its repeated and prolonged thoracic efforts, which contain incontrovertibly more power. Again, a value of one-half was chosen because it represents a significant difference between the PSDs. If only central events are present in the 300-s window of the signal, the computed threshold would be too low, and the algorithm would not be able to detect central apneas. If this happens, a support branch of the algorithm computes the average PSD in the 10 s before each apnea event, storing it in the variable PowerBreathing. This is shown in Figure 19. The power value of each sleep apnea event (PowerApnea) is compared to the PSD associated to the respiration occurring before the same event (PowerBreathing). The check is performed with the condition PowerApnea<PowerBreathing. If this condition is verified, the sleep apnea is central. This portion of the algorithm is activated only if no central apneas are detected in the 300-s window, which can happen if all the apnea events are obstructive.

## 8. Algorithm for the Position Classification

**Figure 20:**
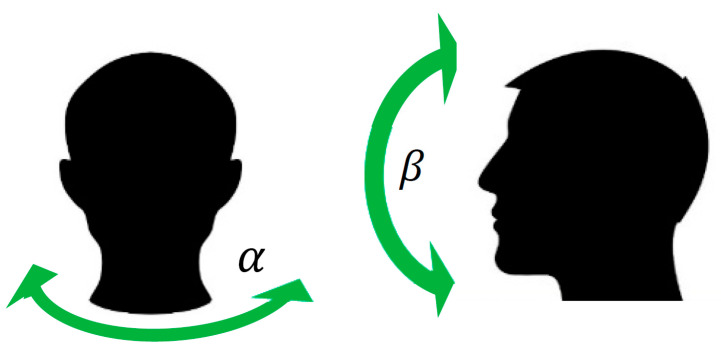
Head rotation measured by α and head inclination measured by β.

In this section, we described how the system can identify the patient position during sleep. We used the raw accelerometer signals from x, y, and z axes, acquired with a sampling frequency of 16 Hz. This test does not require a real patient, since it is just a measure of orientation and, therefore, it is not related to the pathology. The three raw accelerations alone are not enough to distinguish some of the positions from one another and must be further processed. That is why we introduce the 2D Cordic algorithm for the position classification. This algorithm [57] is used in the computation of inclination angles—let us call them α and β—which give information about the inclination of the sensor and, consequently, about the sleep position. In this case, the sensor is placed on the nose, so α and β give information about, respectively, the rotation and inclination of the head (see Figure 20).

**Figure 21:**
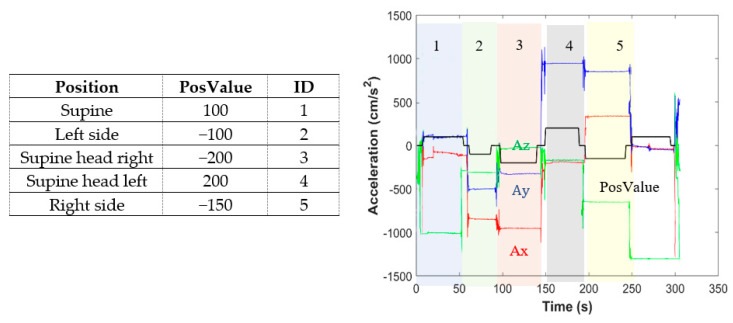
PosValues overlapped on the three raw acceleration signals and. Right: PosValues according to position.

The classification of the subject sleep position is performed using the average acceleration along the three axes and the two mentioned angles. There are a lot of possible positions during sleep, and using just one accelerometer, this algorithm can classify five of them: supine, left side, right side, supine with head turned left, and supine with head turned right, using five different thresholds. We avoided prone positions, since the patient could not be prone while wearing MORFEA. The algorithm outputs a discrete value (PosValue), which depends on the actual position. PosValues are listed in Figure 21. As an example, in the same figure, a test on a control subject is displayed. The curve of PosValues in time is drawn with bold black line together with the three acceleration values. The algorithm successfully classified 100% of the positions tested.

## 9. Conclusions

We proposed a wearable wireless device called MORFEA for the home monitoring of sleep-related breathing disorders, which can be comfortably positioned on the nasal septum during the night. Its performances were tested on an hospitalized patient (and on control subjects for thoraco-abdominal movements detection and body position identification). The patient was wearing our integrated system on the nasal septum and contemporarily performed the recommended respiratory polygraphy along the whole night. On the basis of these results, MORFEA seems potentially able to perform the following: (1) detect any type of apnea and their timing; (2) detect the chest respiratory efforts and thus paving the way for a valid method to distinguish the obstructive and central types; (3) grade SRBD through the AHI parameter; and (4) identify the patient body position during the sleep. MORFEA uses integrated PPG to study the respiration and an accelerometer to monitor movements. Positioning MORFEA on the nasal septum is a strength since it allows for maximum PPG sensitivity to airflow modulation and for an excellent sensitivity of the accelerometer to thoraco-abdominal movements at the same time. MORFEA records and transmits data in streaming mode via Bluetooth Low Energy to an external elaborator. Algorithms have been developed for the apnea detection (using only the PPG), apnea type classification (using the fusion of PPG and inertial signals) and body position identification (using only the accelerometer).

The PPG sensors worked as intended. We achieved a sensitivity of 89% and a precision of 93% in the apnea detection, compared to type III HSAT. We obtained such excellent detection performance by the PPG only thanks to the signal modulation given by the specific sensor position adopted. This is innovative with respect to the literature.

The AHI parameter measured by MORFEA was in extraordinary agreement with the standard nocturnal cardio-pulmonary monitoring, even if MORFEA is still not able to distinguish between an apnea and a hypopnea, since it classifies both as apneas. This does not alter the AHI calculation, since the event count will be the same. In the future, we are planning to address hypopnea recognition with the aid of a new and smarter version of MORFEA.

A method based on the fusion between PPG and accelerometer was proposed to distinguish the obstructive apnea type from the central one. It is based on the detection of respiratory efforts through the accelerometer in those time intervals (detected by the PPG) featuring an airflow reduction. This method was validated on control subjects who performed repeated thoraco-abdominal movements in several different lying down positions, since the accelerometer was unavailable at the time of the patient test. These preliminary results on control subjects are very encouraging, given that the accelerometer was able to detect even the least movements in all the performed tests. However, it should be pointed out that this result is not definitive and needs to be proven on real patient, since testing all the parameters together in a natural sleeping setting is crucial because sleeping has unique pathophysiological features and the algorithms might have different responses. Regarding body position, we got a 100% correct detection.

As a final remark, we wish to point out that all the results resumed above have been achieved with a single low-power, compact, unobtrusive, wearable, and fully wireless device placed on the patient nasal septum, whose battery guaranteed 9 h acquisition.

This makes our device potentially useful for screening sleep-related breathing disorders.

## Figures and Tables

**Table 1:** MORFEA performances.

Total Apneas	False Negatives	Sensitivity	False Positives	Precision	AHI (HSAT)	AHI (MORFEA)
545	62	88.6%	37	92.9%	102	104

## Abbreviations

SPI	Serial Peripheral Interface
I²C	Inter-Integrated Circuit
UART	Universal Asynchronous Receiver–Transmitter
CMOS	Complementary Metal Oxide Semiconductor
ICT	Information Communication Technology
RF	Radiofrequency
BPAP	Bilevel Positive Airway Pressure
PPG	Photoplethysmography
SPO_2_	Blood Oxygen Saturation
ECG	Electrocardiogram
OSA	Obstructive Sleep Apnea
CSA	Central Apnea-Hypopnea
SRBD	Sleep-Related Breathing Disorders
PSG	Polysomnography
CPAP	Continuous Positive Airway Pressure
GSR	Galvanic Skin Response
MEMS	Microelectromechanical Systems
LED	Light Emitting Diode
ANS	Autonomic Nervous System
FDA	Food and Drug Administration
PSD	Power Spectral Density
PWA	Pulse Wave Amplitude
CVD	Cardiovascular Disease
DC	Direct Component (Direct Current is the real meaning, in electronics)
AC	Alternate Component (Alternate Current is the real meaning, in electronics)
